# Overcoming inertia in anticoagulant reversal: the current evidence gap and need for critical assessment of factor replacement strategies

**DOI:** 10.3389/fcvm.2026.1895868

**Published:** 2026-10-06

**Authors:** Charles E. Mahan, Jessica Grandoni, Nikolaos Tsaftaridis, Ralph Riello, Gabriel V. Fontaine, Kyle A. Hultz, Brandon Cave, Adam J. Singer, W. Frank Peacock, Nirav Patel, Arthur Allen, James M. Williams, Christopher W. Baugh, Alex C. Spyropoulos, Mark Goldin

**Affiliations:** 1 College of Pharmacy, University of New Mexico Health Sciences Center, Albuquerque, NM, United States; 2 Anticoagulation Clinic, Dana-Farber Cancer Institute, Boston, MA, United States; 3 Feinstein Institutes for Medical Research, Northwell Health, Manhasset, NY, United States; 4 Department of Internal Medicine, Yale School of Medicine, New Haven, CT, United States; 5 Department of Pharmacy, Intermountain Health, Salt Lake City, UT, United States; 6 Department of Pharmacy, Memorial Hospital West, Pembroke Pines, FL, United States; 7 Department of Veteran Affairs, West Palm Beach VA Health Care System, West Palm Beach, FL, United States; 8 Department of Emergency Medicine, Center for Excellence in Research and Clinical Trials, Renaissance School of Medicine at Stony Brook University, Stony Brook, NY, United States; 9 Henry JN Taub Department of Emergency Medicine, Baylor College of Medicine, Houston, TX, United States; 10 Departments of Neurology and Internal Medicine, Harbor-UCLA Medical Center and Internal Medicine University of California Irvine, Long Beach, CA, United States; 11 Ambulatory Care Pharmacy, Anticoagulation, and Thrombosis Service, VA Salt Lake City Health Care System, Salt Lake City, UT, United States; 12 Texas Tech University Health Sciences Center, Meritus Health, Baltimore, MD, United States; 13 Department of Emergency Medicine, Brigham and Women's Hospital, Harvard Medical School, Boston, MA, United States; 14 The Donald and Barbara Zucker School of Medicine at Hofstra/Northwell, Institute of Health System Science, The Feinstein Institutes for Medical Research, Elmezzi Graduate School of Molecular Medicine, Northwell Health, New York City, NY, United States; 15 Donald and Barbara Zucker School of Medicine at Hofstra/Northwell Health, New York City, NY, United States

**Keywords:** andexanet, anticoagulant, antidote, bleeding, clinical trials, concentrated clotting factors, levels of evidence, prothrombin complex concentrates

## Abstract

**Introduction:**

Direct oral anticoagulants (DOACs) include the factor Xa inhibitors (FXaI) apixaban and rivaroxaban and were approved in the United States and European Union in approximately 2010. Three- and four-factor prothrombin complex concentrates (3F-PCC and 4F-PCC) have been available in the US and EU since 1986 and 2005 for repletion of factors in patients anticoagulated with vitamin K antagonists. Despite their long-standing availability, no large, well-designed prospective clinical trials have been conducted to assess efficacy and safety of PCCs in bleeding patients anticoagulated with DOACs. Although off-label use of PCCs for bleed management of DOAC patients began before the availability of the specific reversal agents idarucizumab and andexanet, utilization increased substantially after antidote approval despite a paucity of evidence. The ANNEXA-I trial compared andexanet with usual care and was terminated early due to superior hemostatic efficacy of andexanet. Heterogeneous interpretation of results underscores significant methodological challenges to performing definitive anticoagulant reversal studies in complex populations at high-risk for mortality.

**Methods:**

Authors identified publications through prior guidance statements, guidelines, review articles, PubMed, and ClinicalTrials.gov. We provide a narrative review of the history of PCCs, other clotting factor concentrates, and the target-specific reversal agent, andexanet for FXaI-related bleeding, in the context of warnings issued by the 2021 Congressional Research Service Report on Off-Label Use. We discuss limitations in regulatory and post-marketing mechanisms for evaluating widespread off-label use. We present a framework to interpret limitations of available real-world evidence on off-label use of 4F-PCC for FXaI-related bleeding, along with clinical practice guideline recommendations.

**Conclusion:**

While target-specific reversal strategies are supported by generally higher quality evidence than non-specific agents, concerns remain about thrombotic complications, functional outcomes, cost-effectiveness, and implementation. Future well-designed, adequately powered, randomized studies should address these concerns to aid decision-making across varied patient bleeding profiles.

## Introduction

The development of direct oral anticoagulants (DOACs) fundamentally shifted the global paradigm for oral anticoagulation (OAC) management. Vitamin K antagonists (VKAs) were previously the only OAC available in the United States (US) since the initial Food and Drug Administration (FDA) approval of warfarin in 1954 (1). Dabigatran, an oral direct thrombin inhibitor (DTI), was approved in the US in 2010, followed by the oral factor Xa inhibitors (FXaIs) rivaroxaban in 2011, apixaban in 2012, edoxaban in 2015, and betrixaban in 2017 (2–6). Indications vary by agent and include reducing the risk of stroke and systemic embolism for patients with non-valvular atrial fibrillation (NVAF), venous thromboembolism (VTE) prevention in a variety of populations, VTE treatment in the acute phase or for extended risk reduction, peripheral arterial disease, and coronary artery disease. Although the lack of a specific reversal agent was initially a barrier to adoption, FXaIs overtook warfarin for new anticoagulant prescriptions as early as 2014 (7). See Figure 1, which depicts a simplified coagulation cascade and common anticoagulants, and Figure 2, which gives a basic timeline of select anticoagulants and reversal agents since approximately 1936. Preferential DOAC use has further increased over time due to an improved safety profile compared with warfarin, conferring approximately half the risk of intracranial hemorrhage (ICH) (8). Additional reasons for the rise in DOAC use include improved patient adherence, less cumbersome monitoring, shared decision-making and patient preference, and reduced costs as DOACs become generic.

**Figure 1:**
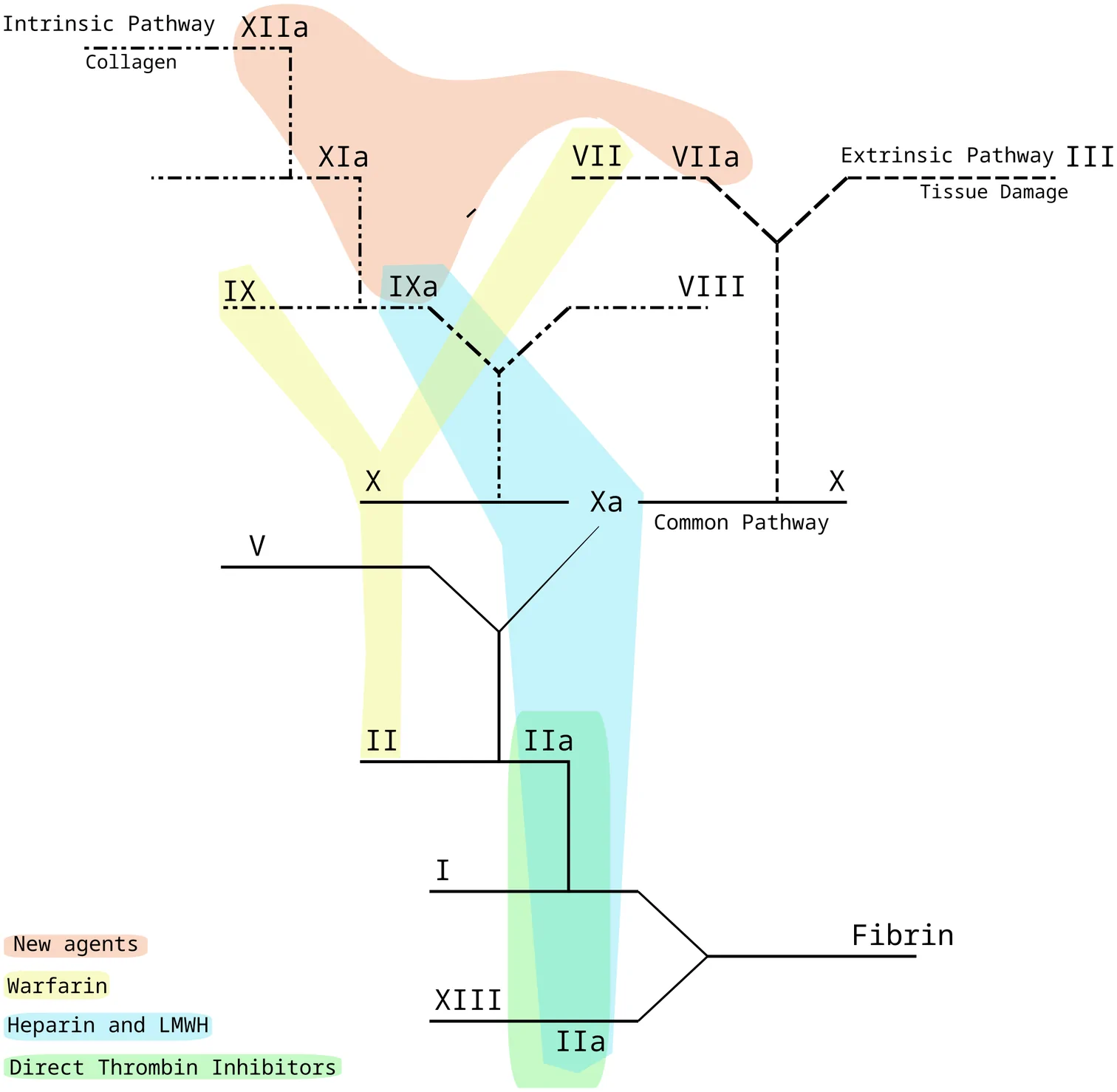
Various anticoagulants and the coagulation cascade. This simplified diagram of the coagulation cascade shows schematically the target proteins of common anticoagulants discussed in this review.

**Figure 2:**
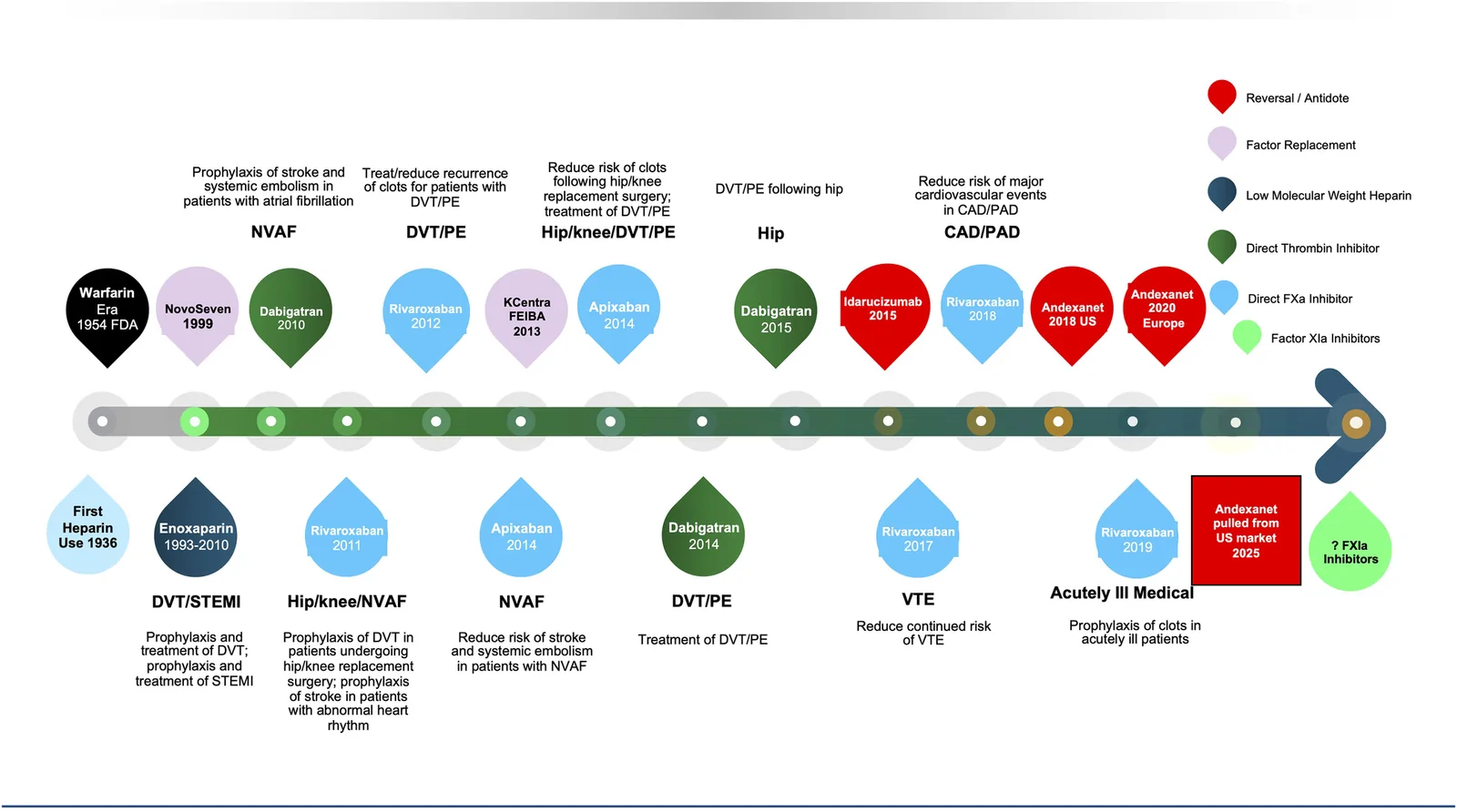
Anticoagulants and reversal agents introduced since 1936. This timeline highlights the introduction of commonly prescribed anticoagulants and their indications, as well as reversal agents discussed in this review.

To address the urgent unmet need to restore hemostasis in the setting of life-threatening DOAC-related bleeding, pharmaceutical manufacturers began developing target-specific reversal agents. Prior to the approval of specific antidotes, the large pivotal DOAC trials were conducted without specific reversal protocols. Thus, clinicians utilized a multitude of non-specific or supportive measures to stabilize bleeding patients with recent DOAC administration. The Anticoagulation Forum noted in 2016 that the quality of evidence for DOAC-related bleeding management with a factor replacement strategy was very low, suggesting that withholding prothrombin complex concentrate (PCC) in this population may be reasonable (9). In the US, 4-factor prothrombin complex concentrate (4F-PCC) was approved by the FDA in late 2012 for the reversal of warfarin in patients with acute major bleeding in conjunction with phytonadione (10). 4F-PCC (Kcentra® and Balfaxar® or Octaplex®) contains coagulation Factors II, VII, IX, X, proteins C and S, as well as heparin, purportedly to prevent thrombosis from any portion of activated factors. 4F-PCC exogenously replaces vitamin K-dependent clotting factors depleted in warfarin-treated patients. Factor eight inhibitor bypassing activity [FEIBA; or activated prothrombin complex concentration (aPCC)], which also contains Factors II, IX, and X and activated Factor VII (i.e., VIIa), was initially approved by the FDA in 1986 and, in 2013, expanded for prophylactic treatment of Hemophilia patients (11). Before the approval of idarucizumab in 2015 and andexanet alfa in 2018, only case reports, limited case series, and observational studies had been published describing the potential role of various coagulation factor concentrates in the treatment of DOAC-related bleeding (12–16).

Major professional society guidelines have provided varying recommendations for FXaI reversal. The American College of Cardiology expert consensus pathway recommends andexanet, with PCC as a less preferred alternative if andexanet is unavailable (17). The International Society of Thrombosis and Haemostasis notes the lack of rigorous studies of PCC for DOAC reversal, but also acknowledges concerns about andexanet, particularly the question of whether decreases in hematoma expansion in ICH (the best studied indication) correlate with important clinical outcomes (18). In contrast, the 2022 American College of Gastroenterology-Canadian Association of Gastroenterology recommends against andexanet due to the lack of a control group in ANNEXA-4, along with generally limited data on gastrointestinal bleeding and cost concerns although the cited cost was outdated and at least 2 to 4 times the cost of andexanet by the time of publication of these guidelines. They also recommended against use of PCC in any DOAC patients hospitalized for acute GI bleeding but made a nuanced statement that andexanet “could be considered in the setting of life-threatening GI bleeding in hospitalized patients who have taken apixaban or rivaroxaban within the past 24 h.” (19) For patients taking DOACs within 24 h and presenting with life-threatening acute lower GI bleeds not responding to resuscitation measures and anticoagulation cessation, the 2023 American College of Gastroenterology Guideline Update recommended the target-specific reversal agents, idarucizumab and andexanet, for treatment while stating “there is no definitive role for PCC in reversal of factor Xa inhibitors.” (20) The most prudent bleed management strategy is now particularly uncertain given the voluntary withdrawal of andexanet alfa from the US market in late 2025, although it remains available in EU markets under conditional approval. Factor replacement strategy was suggested as a second choice in some guidelines or expert consensus statements (18, 19, 21–23).

In 2020, approximately 301,000 patients presented to US emergency departments for bleeding due to OAC, with 200,000 of these due to DOACs. With increasing DOAC use since, DOAC-related major bleeds are anticipated to rise, with an estimated 30,000–40,000 deaths occurring from these bleeds annually (24). The concept of “preventable bleeding” and preventable deaths from bleeding was discussed in the 2014 US National Action Plan for Adverse Drug Event Prevention (25, 26). Recent 2024 US data estimates that between 6,783 to 18,284 preventable deaths occur annually due to the lack of target-specific reversal agents used in patients treated with FXaIs who experience a major bleeding event (27). Therefore, we are compelled to provide the most effective and safest bleed management strategies to improve patient outcomes, including mortality and long-term morbidity, guided by well-designed, well-conducted studies (28). This narrative review will form the first article in a series devoted to DOAC reversal. Here, we will discuss:

1. General definitions of reversal agents and antidotes, and their relation to factor replacement
2. Historical examples of the temporality of anticoagulant introduction and the advent of associated reversal agents
3. The need for additional studies and particular outcome measures

Subsequent reviews in this series will present:

1. Pivotal studies on target-specific reversal agents, including ANNEXA-A, ANNEXA-R, ANNEXA-4, and ANNEXA-I, as well as REVERSE-AD
2. Observational data comparing PCC and Andexanet for reversal of FXaI-related bleeding, as well as real-word evidence studies
3. Comprehensive assessment of ICH management at the health system level

A further article in this series will present original research estimating annual preventable deaths in patients with FXaI-associated bleeding.

### Methodology for literature searches

We performed a literature search of the PubMed and MEDLINE databases for relevant articles including *in vitro* studies, *in vivo* studies, guidelines, randomized control trials, clinical trials, larger observational studies, meta-analyses, and systematic reviews between 2008 and 2026. The following keywords and MeSH terms were used: “Major Bleeding”, “Intracranial Bleeding”, “Intracranial Hemorrhage”, “Hemorrhage”, “Surgical Bleeding”, “Bleeding Reversal”, “Antidote”, “Hemostasis”, “Andexanet Alfa”, “Prothrombin Complex Concentrate”, “Direct Oral Anticoagulant”, “Apixaban”, “Rivaroxaban”, “Dabigatran” and “Factor Xa inhibitor”. Observational studies and retrospective reviews with less than 50 patients were excluded. We utilized the literature search above to identify pertinent guidelines, guidance statements, position statements, and health care entity guidance. We also searched for completed, ongoing and terminated clinical trials on clinicaltrials.gov and utilized the Google search engine to minimize non-capture of pertinent documents. Observational studies, prospective or retrospective, may have been excluded if chart review, as compared to preconstructed databases, was utilized to gather individual patient information if it did not adhere to published minimal recommended standards for chart review extraction. In addition, studies may have been excluded if patients could not be confirmed to be anticoagulated or the last known ingestion of anticoagulant was unknown.

We also conducted PubMed and MEDLINE searches on the definitions of antidote and reversal agent, for each section and subsection of this 6-part themed series, and reviewed both anticoagulant, andexanet, idarucizumab, prothrombin complex concentrate, and other FDA drug package inserts for drugs discussed within. A table of clinical trials and studies discussed as well a separate table of guidelines, guidance statements, positions statements, and health care entity statements will be included with upcoming sections. More weight was given for studies conducted in North America or Europe and those that were registered with clinicaltrials.gov as compared to studies that were not registered. While we utilized a formal evidence hierarchy of placing more weight for instance on systematic reviews, meta-analyses, Cochrane reviews followed by randomized controlled trials, we did not undertake a complete and exhaustive systematic review of all published literature as the sheer volume would be overly expansive; thus, inclusion of all references would be beyond the scope and space of this narrative review.

### Antidotes and reversal agents vs factor replacement

Certain clinical scenarios warrant the emergent removal or reversal of a drug to rapidly neutralize activity, informed by pharmacologic properties and mechanism of action. By convention, nomenclature for reversal agent dictates that the pharmacologic effects of another medication must be substantially counteracted, with important practical implications for anticoagulants, antiplatelets, and other narrow-therapeutic index agents (29, 30). Antidotes, more specifically, counteract the effects of a poison (31) via four basic mechanisms: (a) decreasing the active toxin level, (b) blocking the site of action of the toxin, (c) decreasing the toxic metabolites, or (d) counteracting the effects of the toxin itself (32). These four primary mechanisms would presumably alter the toxin load and/or the duration of exposure.

The rationale for the off-label use of concentrated factor replacement strategies, predominantly 3F- or 4F-PCC, for DOAC-associated bleeding events is to increase multiple coagulation factor concentrations (i.e., factor II, VII, IX, and X), which is postulated to overwhelm coagulation potential above an uncertain inhibition threshold, thereby theoretically restoring hemostasis (33). Because PCC and other factor replacement strategies do not affect serum FXaIs levels, do not interfere with the site of action, and do not decrease toxic FXaIs metabolites, the only possible satisfactory antidote criteria would be to (d) counteract the effects of FXaIs.

While not a global measure of hemostasis, anti-FXa activity is an accepted pharmacodynamic marker of factor Xa inhibitor activity and has been used as a surrogate endpoint in regulatory evaluation of andexanet alfa. Not surprisingly, concentrated factor replacement strategies have shown little effect on anti-FXa levels in trials that evaluated this endpoint (15, 34–39). In 2016, a prospective, multicenter, observational study, called RASUNOA, took place in patients with DOAC-related non-traumatic ICH. PCCs demonstrated no effect on substantial hematoma expansion [43% [12 of 28] for PCC vs 29% [5 of 17] for no PCC, *P* = 0.53] or on the occurrence of having an unfavorable outcome [modified Rankin Scale (mRS) score, 3–6] (OR,1.20; 95% CI, 0.37–3.87; *P* = 0.76) (13). Additionally, 4F-PCC has not been observed to have an *in vitro* effect on endogenous thrombin potential or peak thrombin generation in the presence of rivaroxaban or apixaban until serum levels are reduced to thresholds below what clinicians would normally reverse (40). Further, it appears that 4F-PCC would require the addition of factor V concentrate to possibly be effective in patients anticoagulated with FXaI; however, factor V concentrate is not commercially available in either the US or the EU (33). Thus, with respect to FXaI bleed management, 4F-PCC and other factor-repletion strategies do not meet consensus definitions of antidotes or reversal agents in this population. Moreover, PCC was never incorporated into routine practice for the management of major bleeding due to the DTI, dabigatran–even before the approval of idarucizumab. Similar to andexanet studies, REVERSE-AD studied idarucizumab for the reversal of dabigatran and used the surrogate endpoints of ecarin clotting time and thrombin time to assess reversal efficacy.

Thus, while not clearly meeting criteria as an antidote or reversal agent as outlined above, 4F-PCC outpaced its labeled indication for warfarin reversal–more than doubling Kcentra® revenue growth from 7%–16% year-over-year from 2021 to 2025 (41–45) – despite VKA use steadily declining across the US since 2013 (7, 24, 46–48). Although DOAC-related hemorrhage generally confers better prognosis than warfarin-related bleeding, recent retrospective studies have also paradoxically shown that patients with off-label use of PCC have an approximately 40%–50% higher mortality rate as compared to patients who had PCC used for on-label purposes (49, 50).

These notable discrepancies should serve as a call to action for professional organizations to examine off-label use of 4F-PCC for FXaI bleeding. Indeed, it is not known whether placebo confers less risk to healthy volunteers anticoagulated with FXaI, let alone bleeding patients prescribed FXaIs for chronic thromboembolic disorders, as the requisite trials have not been conducted (12–14, 51). Methodological limitations of currently published 4F-PCC studies have precluded establishing whether enrolled patients were therapeutically anticoagulated at baseline. Given the short half-life of FXaIs and undocumented adherence rates, it is reasonable to conclude that many patients in 4F-PCC FXaI bleeding studies may not have required reversal or factor replacement at all. Thus, in a small subset of 4F-PCC studies to date, investigators may simply be observing the usual effects of endogenous hemostatic mechanisms or the physiologic restoration of hemostasis as FXaIs are eliminated, with a surplus of exogenous factor repletion (12, 14, 40, 51, 52). Substantial declines in warfarin prescribing obscure the increase in PCC use, but nonetheless suggest that as much as ≥80% of 4F-PCC use may be off-label for FXaI-related bleeds. Due to the lack of phase III data for PCC in this population demonstrating efficacy and safety, no discernible effect of PCC on anti-FXa activity, and absence of rigorous populational-level follow-up after hospital discharge, there is concern regarding the wide-spread off label use of PCC and its long-term ramifications (53). Given the totality of evidence and the lack of PCC meeting the minimum definition of an antidote or reversal agent, factor repletion strategies should be reconsidered as a treatment option in this population.

### Theme of lack of reversal upon anticoagulant approval

Since the first anticoagulant, unfractionated heparin, through the serendipitous discovery of warfarin, the advent of DOACs, and perhaps next the emergence of Factor XIa inhibitors (FXIaI), there has never been a simultaneous market debut of a new anticoagulant with its antidote or reversal agent (54). Over the last century, the net clinical benefit of anticoagulation across indications has been driven primarily by meaningful reductions in thromboembolic risk, despite the inherent potential to cause clinically relevant bleeds without a proven reversal strategy. It is crucial to recall that DOACs have demonstrated consistent safety benefits over warfarin with respect to bleeding risk during clinical development–well before Kcentra® was initially FDA-approved. There are also parallels with the development of other antithrombotic agents, including antiplatelet agents such as aspirin, clopidogrel, ticagrelor, and prasugrel. To date, none of these antiplatelets agents has an approved target-specific reversal agent; however, the FDA has recently accepted bentracimab's Biologics License Application and is reviewing it for ticagrelor reversal under priority review. This contemporary example demonstrates a 15-year gap in the availability of antithrombotics and a potential antidote for these therapies.

Following the discovery of heparin by Howell in 1922, the FDA granted clinical approval in the US for bovine and porcine heparin in 1939 and 1942, respectively. The first documented use of injectable heparin in a human subject occurred in 1937 in Canada, although two Swedish doctors, Hedenius and Wilander, arguably injected heparin in the same period (likely 1935 or 1936) to assess its effects (54). While never approved by the FDA for human use, hexadimethrine bromide (Polybrene) was used as the first original reversal agent for heparin starting in the early 1950's in areas such as open-heart surgery and into the 1960s, although the drug had no human regulatory approval (55). Due to reports that hexadimethrine caused nephrotoxicity, use fell out of favor, and it was replaced by protamine sulfate in 1969 (54, 56, 57). With the introduction of low molecular weight heparins (LMWH) in the 1990's, protamine sulfate was also used to reverse LMWH, although it is only approximately 60% effective (58). A fully effective reversal for LMWH has not yet been brought to market, although andexanet appears to be effective in reversing both LMWH and fondaparinux (59). To date, no specific reversal agent has been brought to market to reverse fondaparinux.

Dicumarol was the first VKA discovered, and was FDA-approved for use in the mid-1940s (60, 61). Warfarin, developed and introduced as an efficient rodenticide by Karl Link, was later approved by the FDA in 1954 (60, 62). The Danish biochemist Henrik Dam was credited with the discovery of the vitamin K antidote, while the American biochemist Edward A. Doisy independently elucidated the chemical structure of the naphthoquinone ring and the nature of the vitamin; thus, both men shared the Nobel Prize in Medicine in 1943 (61–64). Phytonadione is believed to have first been used in 1951 as an antidote for dicumarol overdose (62). However, it should be noted that phytonadione is not an immediate antidote, but is considered for semi-urgent reversal of warfarin by reenabling hepatic synthesis of vitamin K-dependent clotting factors (II, VII, IX, X) and endogenous anticoagulants Protein C and S (61, 65). Even with the most rapidly absorbed intravenous phytonadione, onset is estimated to be approximately two hours, with peak effect occurring around 12 h; oral forms have longer onset and peak times (65). Although FEIBA was made available in the US in 1986 and in Europe in 1976, it was not marketed as a warfarin reversal agent. It was only in 2013 in the US and 1996 in the EU that 4F-PCC became available as a true antidote for VKAs, 4–5 decades after their introduction.

DOACs entered the global anticoagulation market nearly one half-century after warfarin, boasting superior bleeding safety even in the absence of an approved reversal agent. Despite this, an approved antidote to reverse bleeding related to FXaI use remained a formidable barrier to early uptake. Clinicians, however, eventually realized that DOACs were at least as effective as well-managed warfarin for most thromboembolic indications, yet, as a class, significantly safer from a bleeding standpoint (8). This was especially true for the most concerning major bleeding events, ICH and fatal bleeds, with pivotal trials of the individual agents consistently demonstrating an incidence approximately 50% lower than VKAs (8, 66, 67). Critically, national trends for new anticoagulant prescriptions in the US began favoring DOACs over warfarin as early as 2014 (7, 46), long before published evidence for any DTI or FXaI reversal strategy.

With dabigatran specifically approved for use in the US in 2010, idarucizumab was first approved in 2015 under an accelerated pathway and gained full approval in 2018, supported by the full results of the REVERSE-AD trial in both medical and surgical populations (68, 69). However, it should be noted that there was no comparator group for idarucizumab***,*** no known hemostatic efficacy rates for ICH, and only surrogate endpoints were used to determine clinical efficacy. Importantly, regulatory agencies did not require further post-marketing analyses for idaucizumab to remain licensed for dabigatran reversal. By contrast, initial approval of andexanet alfa was contingent upon the successful completion of a randomized controlled trial versus usual care (UC) in high-risk patients with ICH despite FXaI already being the primary anticoagulants used in the US and EU.

The pursuit of FXIaI for anticoagulation also follows the same theme, with potential market introduction contingent on regulatory approval and the absence of a reversal agent. Reversal studies are also more difficult to design, undertake, and execute, given that patients with major bleeding are a very high-risk and heterogeneous group to treat. Nevertheless, current evidence supports a clear pharmacologic distinction between target-specific reversal agents and non-specific factor replacement strategies. For Factor Xa inhibitor-associated major bleeding, PCCs do not directly neutralize drug activity and remain supported primarily by observational and indirect evidence. And, while available data for target-specific reversal agents is typically higher quality, careful interpretation is needed, given concerns about thrombotic complications. Future adequately powered randomized trials and high-quality post-marketing studies should evaluate hemostatic efficacy, thrombotic events, mortality, and particularly functional patient outcomes, in addition to cost-effectiveness, and implementation feasibility across bleeding phenotypes. Until such data are available, reversal strategies should be selected through individualized, context-and patient-specific multidisciplinary decision-making.

## References

[1] US Food and Drug Administration. Available online at: https://www.fda.gov/about-fda/fda-history-exhibits/drug-therapeutics-regulation-us (Accessed April 9, 2024).

[2] Dabigatran FDA Package Insert. Available online at: https://www.accessdata.fda.gov/drugsatfda_docs/label/2011/022512s007lbl.pdf (Accessed May 24, 24).

[3] Rivaroxaban FDA Package Insert. Available online at: https://www.janssenlabels.com/package-insert/product-monograph/prescribing-information/XARELTO-pi.pdf (Accessed May 24, 24)

[4] Apixaban FDA Package Insert Available online at: https://packageinserts.bms.com/pi/pi_eliquis.pdf (Accessed May 24, 24).

[5] Edoxaban FDA Package Insert Available online at: https://www.accessdata.fda.gov/drugsatfda_docs/label/2015/206316lbl.pdf (Accessed May 24, 24).

[6] Betrixaban FDA Package Insert Available online at: https://www.accessdata.fda.gov/drugsatfda_docs/label/2020/208383s007lbl.pdf (Accessed May 24, 24).

[7] Mahan CE. Practical aspects of treatment with target specific anticoagulants: initiation, payment and current market, transitions, and venous thromboembolism treatment. J Thromb Thrombolysis. (2015) 39(3):295–303. 10.1007/s11239-014-1164-4 25605686

[8] Ruff CT Giugliano RP Braunwald E Hoffman EB Deenadayalu N Ezekowitz MD. Comparison of the efficacy and safety of new oral anticoagulants with warfarin in patients with atrial fibrillation: a meta-analysis of randomised trials. Lancet. (2014) 383(9921):955–62. 10.1016/S0140-6736(13)62343-0 24315724

[9] Burnett AE Mahan CE Vazquez SR Oertel LB Garcia DA Ansell J. Guidance for the practical management of the direct oral anticoagulants (DOACs) in VTE treatment. J Thromb Thrombolysis. (2016) 41(1):206–32. 10.1007/s11239-015-1310-7 26780747 PMC4715848

[10] Kcentra FDA Package Insert Available online at: https://www.fda.gov/media/85512/download Accessed 5-24-2024

[11] FEIBA FDA Package Insert Available online at: https://www.shirecontent.com/PI/PDFs/FEIBA_USA_ENG.pdf (Accessed May 24, 24).

[12] Majeed A Ågren A Holmström M Bruzelius M Chaireti R Odeberg J. Management of rivaroxaban- or apixaban-associated major bleeding with prothrombin complex concentrates: a cohort study. Blood. (2017) 130(15):1706–12. 10.1182/blood-2017-05-782060 28835439

[13] Purrucker JC Haas K Rizos T Khan S Wolf M Hennerici MG. Early clinical and radiological course, management, and outcome of intracerebral hemorrhage related to new oral anticoagulants. JAMA Neurol. (2016) 73(2):169–77. 10.1001/jamaneurol.2015.3682 26660118

[14] Schulman S Gross P Ritchie B Nahirniak S Lin Y Lieberman L. Prothrombin Complex concentrate for Major bleeding on factor Xa inhibitors: a prospective cohort study. Thromb Haemost. (2018) 118(5):842–51. 10.1055/s-0038-1636541 29564837

[15] Song Y Wang Z Perlstein I Wang J LaCreta F Frost RJA. Reversal of apixaban anticoagulation by four-factor prothrombin complex concentrates in healthy subjects: a randomized three-period crossover study. J Thromb Haemost. (2017) 15(11):2125–37. 10.1111/jth.13815 28846831

[16] Gerner ST Kuramatsu JB Sembill JA Sprügel MI Endres M Haeusler KG. Association of prothrombin complex concentrate administration and hematoma enlargement in non-vitamin K antagonist oral anticoagulant-related intracerebral hemorrhage. Ann Neurol. (2018) 83(1):186–96. 10.1002/ana.25134 29314216

[17] Tomaselli GF Mahaffey KW Cuker A Dobesh PP Doherty JU Eikelboom JW. 2020 ACC expert consensus decision pathway on management of bleeding in patients on oral anticoagulants: a report of the American College of Cardiology solution set oversight committee. J Am Coll Cardiol. (2020) 76(5):594–622. 10.1016/j.jacc.2020.04.053 32680646

[18] Levy JH Shaw JR Castellucci LA Connors JM Douketis J Lindhoff-Last E. Reversal of direct oral anticoagulants: guidance from the SSC of the ISTH. J Thromb Haemost. (2024) 22(10):2889–99. 10.1016/j.jtha.2024.07.009 39029742

[19] Abraham NS Barkun AN Sauer BG Douketis J Laine L Noseworthy PA. American College of gastroenterology-Canadian association of gastroenterology clinical practice guideline: management of anticoagulants and antiplatelets during acute gastrointestinal bleeding and the periendoscopic period. Am J Gastroenterol. (2022) 117(4):542–58. 10.14309/ajg.0000000000001627 35297395 PMC8966740

[20] Sengupta N Feuerstein JD Jairath V Shergill AK Strate LL Wong RJ. Management of patients with acute lower gastrointestinal bleeding: an updated ACG guideline. Am J Gastroenterol. (2023) 118(2):208–31. 10.14309/ajg.0000000000002130 36735555

[21] Abraham NS Barkun AN Sauer BG Douketis J Laine L Noseworthy PA. American College of gastroenterology-Canadian association of gastroenterology clinical practice guideline: management of anticoagulants and antiplatelets during acute gastrointestinal bleeding and the periendoscopic Period. J Can Assoc Gastroenterol. (2022) 5(2):100–1. 10.1093/jcag/gwac010 35368325 PMC8972207

[22] Bavalia R Abdoellakhan R Brinkman HJM Brekelmans MPA Hamulyák EN Zuurveld M. Emergencies on direct oral anticoagulants: management, outcomes, and laboratory effects of prothrombin complex concentrate. Res Pract Thromb Haemost. (2020) 4(4):569–81. 10.1002/rth2.12336 32548555 PMC7292661

[23] Singer AJ Abraham NS Ganti L Peacock WF Dark J Ishaq H. Evaluation and treatment of gastrointestinal bleeding in patients taking anticoagulants presenting to the emergency department. Int J Emerg Med. (2024) 17(1):70. 10.1186/s12245-024-00649-7 38822267 PMC11141076

[24] Geller AI Shehab N Lovegrove MC Weidle NJ Budnitz DS. Bleeding related to oral anticoagulants: trends in US emergency department visits, 2016–2020. Thromb Res. (2023) 225:110–5. 10.1016/j.thromres.2023.03.010 37062120 PMC10870325

[25] U.S. Department of Health and Human Services, Office of Disease Prevention and Health Promotion. (2014). National Action Plan for Adverse Drug Event Prevention. Washington, DC. Available online at: https://odphp.health.gov/sites/default/files/2019-09/ADE-Action-Plan-508c.pdf (Accessed April 9, 2024).

[26] Caraballo D Spyropoulos AC Mahan CE. Identifying, monitoring and reducing preventable major bleeds in the hospital setting. J Thromb Thrombolysis. (2013) 36(1):7–13. 10.1007/s11239-012-0819-2 23054465

[27] Mahan CE. Estimate of 2024 preventable deaths in US patients treated with apixaban and rivaroxaban requiring reversal for Major bleeding events. Res Pract Thrombo Haemost. 2024 8(Suppl 2):3. 102504, ISTH 2024 Meeting Abstracts Available online at: https://doiorg/101016/jrpth2024102504 2024; Abstract Presentation/Board Number: PB1000 on June 25, 2024. Available online at: https://www.rpthjournal.org/article/S2475-0379(24)00199-7/fulltext (Accessed September 8, 2026).

[28] Dobesh PP Fermann GJ Christoph MJ Koch B Lesén E Chen H. Lower mortality with andexanet alfa vs 4-factor prothrombin complex concentrate for factor Xa inhibitor-related major bleeding in a U.S. Hospital-based observational study. Res Pract Thromb Haemost. 2023 7(s6):102192. 10.1016/j.rpth.2023.102192 37753225 PMC10518480

[29] Available online at: https://medical-dictionary.thefreedictionary.com/reversal+agent (Accessed April 5, 2024)

[30] Wong M. Reversal agents in sedation and anesthesia practice for dentistry. Anesth Prog. (2022) 69(1):49–58. 10.2344/anpr-69-01-09 35377935 PMC8985463

[31] Available online at: https://www.merriam-webster.com/dictionary/antidote (Accessed April 5, 2024).

[32] Chacko B Peter JV. Antidotes in poisoning. Indian J Crit Care Med. (2019) 23(Suppl 4):S241–S9. 10.5005/jp-journals-10071-23310 32020997 PMC6996653

[33] Brinkman HJM Swieringa F Zuurveld M Veninga A Brouns SLN Heemskerk JWM. Reversing direct factor Xa or thrombin inhibitors: factor V addition to prothrombin complex concentrate is beneficial *in vitro*. Res Pract Thromb Haemost. (2022) 6(3):e12699. 10.1002/rth2.12699 35494506 PMC9036856

[34] Barco S Whitney Cheung Y Coppens M Hutten BA Meijers JC Middeldorp S. *In vivo* reversal of the anticoagulant effect of rivaroxaban with four-factor prothrombin complex concentrate. Br J Haematol. (2016) 172(2):255–61. 10.1111/bjh.13821 26488126

[35] Cheung YW Barco S Hutten BA Meijers JC Middeldorp S Coppens M. *In vivo* increase in thrombin generation by four-factor prothrombin complex concentrate in apixaban-treated healthy volunteers. J Thromb Haemost. (2015) 13(10):1799–805. 10.1111/jth.13115 26270625

[36] Eerenberg ES Kamphuisen PW Sijpkens MK Meijers JC Buller HR Levi M. Reversal of rivaroxaban and dabigatran by prothrombin complex concentrate: a randomized, placebo-controlled, crossover study in healthy subjects. Circulation. (2011) 124(14):1573–9. 10.1161/CIRCULATIONAHA.111.029017 21900088

[37] Levi M Moore KT Castillejos CF Kubitza D Berkowitz SD Goldhaber SZ. Comparison of three-factor and four-factor prothrombin complex concentrates regarding reversal of the anticoagulant effects of rivaroxaban in healthy volunteers. J Thromb Haemost. (2014) 12(9):1428–36. 10.1111/jth.12599 24811969

[38] Levy JH Moore KT Neal MD Schneider D Marcsisin VS Ariyawansa J. Rivaroxaban reversal with prothrombin complex concentrate or tranexamic acid in healthy volunteers. J Thromb Haemost. (2018) 16(1):54–64. 10.1111/jth.13894 29106076

[39] Nagalla S Thomson L Oppong Y Bachman B Chervoneva I Kraft WK. Reversibility of apixaban anticoagulation with a four-factor prothrombin Complex concentrate in healthy volunteers. Clin Transl Sci. (2016) 9(3):176–80. 10.1111/cts.12398 27170068 PMC5351332

[40] Lu G Lin J Bui K Curnutte JT Conley PB. Andexanet versus prothrombin complex concentrates: differences in reversal of factor Xa inhibitors in *in vitro* thrombin generation. Res Pract Thromb Haemost. (2020) 4(8):1282–94. 10.1002/rth2.12418 33313467 PMC7695565

[41] CSL Behring Annual Report 2021, Pages 18-19 & 25, Available online at: https://investors.csl.com/annualreport/2021/20/ (Accessed April 9, 2024).

[42] CSL Annual Report 2022, Available online at: https://investors.csl.com/annualreport/2022/ (Accessed April 9, 2024).

[43] CSL Annual Report 2023, Available online at: https://www.csl.com/-/media/shared/documents/annual-report/csl-annual-report-2023.pdf Pages,s (Accessed April 9, 2024).

[44] CSL Annual Report 2024 Available online at: https://investors.csl.com/pdf/78576f85-4598-455b-80c1-16c391c56445/Results-Presentation.pdf?Platform=ListPage (Accessed January 19, 2026).

[45] CSL Annual Report 2025 Available online at: https://investors.csl.com/pdf/c72789c6-778d-4b88-8657-be40cf97e2b3/CSL-Results-Presentation.pdf?Platform=ListPage (Accessed January 16, 2026)

[46] Barnes GD Lucas E Alexander GC Goldberger ZD. National trends in ambulatory oral anticoagulant use. Am J Med. (2015) 128(12):1300–5 E2. 10.1016/j.amjmed.2015.05.044 26144101 PMC4658248

[47] Iyer GS Tesfaye H Khan NF Zakoul H Bykov K. Trends in the use of oral anticoagulants for adults with venous thromboembolism in the US, 2010–2020. JAMA Netw Open. (2023) 6(3):e234059. 10.1001/jamanetworkopen.2023.4059 36947039 PMC10034573

[48] Navar AM Kolkailah AA Overton R Shah NP Rousseau JF Flaker GC. Trends in oral anticoagulant use among 436 864 patients with atrial fibrillation in community practice, 2011 to 2020. J Am Heart Assoc. (2022) 11(22):e026723. 10.1161/JAHA.122.026723 36346063 PMC9750070

[49] Adkins BD Shaim H Abid A Gonzalez A DeAnda A Jr Yates SG. Four-factor prothrombin complex concentrate use for on-label versus off-label indications: a retrospective cohort study. J Thromb Thrombolysis. 2023 55(1):74–82. 10.1007/s11239-022-02671-z 35699871

[50] Cho BC Jung YH DeMario VM Lau E Podlasek SJ Grant MC. On-label compared to off-label four-factor prothrombin complex concentrate use: a retrospective, observational study. Transfusion. (2019) 59(8):2678–84. 10.1111/trf.15355 31121073

[51] Mahan CE. Reply to “key points to consider when evaluating andexxa for formulary addition”. Neurocrit Care. (2020) 33(1):323–6. 10.1007/s12028-020-01008-z 32572822 PMC7392924

[52] Panos NG Cook AM John S Jones GM Kelly H Choi RK. Neurocritical care society pharmacy study, G. Factor Xa inhibitor-related intracranial hemorrhage: results from a multicenter, observational cohort receiving prothrombin Complex concentrates. Circulation. (2020) 141(21):1681–9. 10.1161/CIRCULATIONAHA.120.045769 32264698

[53] CE Mahan and the FDA, Personal Communication, 5/27/2022; WF Peacock and the FDA, Personal Communication (Accessed November 21, 2024).

[54] Hemker HC. A century of heparin: past, present and future. J Thromb Haemost. (2016) 14(12):2329–38. 10.1111/jth.13555 27862941

[55] Frackiewicz A Kalaska B Miklosz J Mogielnicki A. The methods for removal of direct oral anticoagulants and heparins to improve the monitoring of hemostasis: a narrative literature review. Thromb J. (2023) 21(1):58. 10.1186/s12959-023-00501-7 37208753 PMC10197842

[56] Lillehei CW Sterns LP Long DM Lepley D Jr. Comparative study of polybrene and protamine for heparin neutralization in open heart surgery. Ann Surg. (1960) 151(1):11–6. 14416954 PMC1613338

[57] Ransdell HT Jr Haller JA Jr Stowens D Barton PB. Renal toxicity of polybrene, (hexadimethrine bromide). J Surg Res. (1965) 5:195–9. 10.1016/S0022-4804(65)80086-5 14281435

[58] Hirsh J Raschke R. Heparin and low-molecular-weight heparin: the seventh ACCP conference on antithrombotic and thrombolytic therapy. Chest. (2004) 126(3 Suppl):188S–203. 10.1378/chest.126.3_suppl.188S 15383472

[59] Levy JH Ghadimi K Kizhakkedathu JN Iba T. What's fishy about protamine? Clinical use, adverse reactions, and potential alternatives. J Thromb Haemost. (2023) 21(7):1714–23. 10.1016/j.jtha.2023.04.005 37062523

[60] Available online at: https://www.fda.gov/about-fda/fda-history-exhibits/drug-therapeutics-regulation-us#:∼:text=Following%20clinical%20investigations%20of%20the,%2C%20in%20the%20mid%2D1940s (Accessed May 19, 2025).

[61] Heestermans M Poenou G Hamzeh-Cognasse H Cognasse F Bertoletti L. Anticoagulants: a short history, their mechanism of action, pharmacology, and indications. Cells. (2022) 11(20):3214. 10.3390/cells11203214 36291080 PMC9600347

[62] Link KP. The discovery of dicumarol and its sequels. Circulation. (1959) 19(1):97–107. 10.1161/01.CIR.19.1.97 13619027

[63] Grober U Reichrath J Holick MF Kisters K. Vitamin K: an old vitamin in a new perspective. Dermatoendocrinol. (2014) 6(1):e968490. 10.4161/19381972.2014.968490 26413183 PMC4580041

[64] Shampo MA Kyle RA. Henrik dam–discoverer of vitamin K. Mayo Clin Proc. (1998) 73(1):46. 10.1016/S0025-6196(11)63617-3 9443677

[65] Thigpen JL Limdi NA. Reversal of oral anticoagulation. Pharmacotherapy. (2013) 33(11):1199–213. 10.1002/phar.1270 23606318 PMC3800206

[66] Papp T Kiss Z Rokszin G Fábián I Márk L Bagoly Z. Mortality on DOACs versus on vitamin K antagonists in atrial fibrillation: analysis of the Hungarian health insurance fund database. Clin Ther. (2023) 45(4):333–46. 10.1016/j.clinthera.2023.03.008 37028991

[67] Wu T Lv C Wu L Chen W Lv M Jiang S. Risk of intracranial hemorrhage with direct oral anticoagulants: a systematic review and meta-analysis of randomized controlled trials. J Neurol. (2022) 269(2):664–75. 10.1007/s00415-021-10448-2 33594452

[68] Idarucizumab FDA Approval Available online at: https://www.fda.gov/drugs/accelerated-approval-program/verified-clinical-benefit-non-malignant-hematological-neurological-and-other-disorder-indications (Accessed May 21, 2025).

[69] Pollack CV Jr Reilly PA Van Ryn J Eikelboom JW Glund S Bernstein RA. Idarucizumab for dabigatran reversal - full cohort analysis. N Engl J Med. 2017 377(5):431–41. 10.1056/NEJMoa1707278 28693366

